# Editorial: Mining Scientific Papers, Volume II: Knowledge Discovery and Data Exploitation

**DOI:** 10.3389/frma.2022.911070

**Published:** 2022-06-15

**Authors:** Iana Atanassova, Marc Bertin, Philipp Mayr

**Affiliations:** ^1^CRIT—Université de Bourgogne Franche-Comté, Besançon, France; ^2^Institut Universitaire de France (IUF), Paris, France; ^3^ELICO—Université Claude Bernard Lyon 1, Lyon, France; ^4^GESIS—Leibniz-Institute for the Social Sciences, Cologne, Germany

**Keywords:** text mining, scientific papers, scientometrics, natural language processing, computational linguistics, citation content analysis, academic search

## 1. Introduction

The Research Topic on “*Knowledge Discovery and Data Exploitation*” aims at promoting interdisciplinary research in computational linguistics and in Natural Language Processing (NLP) applied to the fields of Bibliometrics, Scientometrics, and Information Retrieval. It is a follow-up of our previous Research Topic: “*Mining Scientific Papers: NLP-enhanced Bibliometrics*” (Atanassova et al., 2019).

The processing of scientific texts, which includes the analysis of citation contexts but also the task of information extraction from scientific papers for various applications, has been the object of intensive research during the last decade. This has become possible thanks to two factors. The first one is the growing availability of scientific papers in full text and in machine-readable formats together with the rise of the Open Access publishing of papers on online platforms such as ArXiv, Semantic Scholar, CiteSeer, or PLOS. The second factor is the relative maturity of open source tools and libraries for natural language processing that facilitate text processing (e.g., Spacy, NLTK, Mallet, OpenNLP, CoreNLP, Gate, CiteSpace). As a result, a large number of experiments have been conducted by processing the full text of papers for citation context analysis, but also summarization and recommendation of scientific papers.

This Research Topic aims to discuss novel approaches that focus on the processing and exploitation of data extracted from scientific literature. In particular, the possibility to enrich metadata by the full-text processing of papers offers new fields of investigation that are related to the representation of data and the production of knowledge by the aggregation of data from multiple documents. Given the wide range of available techniques, several questions arise in this field: What volume of scientific data should be considered exploitable and allow the production of new knowledge through aggregation? How can knowledge generated from data in scientific articles be represented? What types of data and knowledge can be automatically extracted from scientific articles and how can it be exploited efficiently?

## 2. Papers in This Research Topic

The six papers published in this Research Topic were all reviewed by at least two independent reviewers who have been assigned by the editors.

In the paper “*Language Bias in Health Research: External Factors That Influence Latent Language Patterns”* Valdez and Goodson the authors propose to use topic modeling to study the linguistic properties of abstracts of papers in Health research and predict language bias. The paper analyses the language alterations according to three factors: time, funding sources and nation of origin. The results show that each of these three factors influence the linguistic patterns used in the abstracts of papers.

The paper titled “*Large Scale Subject Category Classification of Scholarly Papers With Deep Attentive Neural Networks”* Kandimalla et al. propose a method for classifying scientific articles based on their abstracts. For this purpose, the authors propose to use a deep attentive neural network (DANN) trained on abstracts obtained from the Web of Science (WoS) and its categories. The results obtained are better than existing approaches based on clustering and citation networks.

The paper “*SYMBALS: A Systematic Review Methodology Blending Active Learning and Snowballing”* van Haastrecht et al. introduce an innovative systematic review methodology, called SYMBALS. SYMBALS blends the traditional method of backward snowballing with the machine learning method of active learning. The authors proved the validity of their method using a replication study with ASReview, where SYMBALS could accelerate the title and abstract screening.

The opinion paper “*Enhancing Knowledge Graph Extraction and Validation From Scholarly Publications Using Bibliographic Metadata”* Turki et al. elaborates on how each type of bibliographic metadata can provide useful insights to enhance the automatic enrichment and fact-checking of knowledge graphs from scholarly publications. The authors explore about research efforts connected to the Bibliometric-enhanced Information Retrieval initiative (Cabanac et al., 2020a,b).

The paper “*Visual Summary Identification From Scientific Publications via Self-Supervised Learning”* Yamamoto et al. builds a novel benchmark data set for visual summary identification from scientific publications, which consists of papers presented at computer science conferences. The authors introduce and evaluated a new self-supervised learning approach to learn a heuristic matching of in-text references to figures with figure captions.

The paper “*NLP4NLP+5: The Deep (R)evolution in Speech and Language Processing”* Mariani et al. continues the series of two papers that were published on the NLP4NLP corpus in our previous Research Topic. This new paper uses similar methods, but adds to the dataset 5 more years of publications, between 2016 and 2020. Research in the field of Speech and Language Processing during these years has been intense and some significant evolution in the Research Topics can be observed. The analysis of the dataset shows that large communities have shifted their research to novel topics such as Neural Networks and Word Embeddings. This, together with the acceleration of the publication process and the growth in the use of language resources, account for some important transformations in this field of research. The authors provide a thorough analysis of the dataset that shows these phenomena.

## 3. Conclusion

The topic of mining scientific papers, and more broadly text mining methods used in the fields of NLP-enhanced Bibliometrics and knowledge discovery, generate much interest from the community. At the moment of publication of this editorial, the two Research Topics on **Mining Scientific Papers** Vol 1 *NLP-enhanced Bibliometrics*^1^ and Vol 2 *Knowledge Discovery and Data Exploitation*^2^ have attracted more than 99,000 and 24,000 views respectively.

The set of papers that were published in the two Research Topics show various methods that were applied to the full text of articles, or their metadata, references and abstracts. The Table 1 presents an overview of all 13 papers that were published. This table shows the variety of topics and areas of applications that were addressed, as well as the objects, corpora and methods that were used (the table scheme was copied from Cabanac et al., 2020b).

**Table 1:** Overview of the articles in the RTs Vol 1/Vol 2.

**Task**	**Area of applications**	**Corpus**	**Objects**	**Methods**
Ermakova et al. (2018)				
Measuring representativeness of abstracts	Environmental sciences	ISTEX	Fulltext	Text classification, text similarity
Meyers et al. (2018)				
Terminology Extraction	Texts and Patents	US patents, Web of Science	Fulltext	Chunking, Reranking
Rodrigues Alves et al. (2018)				
Reference Mining	Arts and Humanities	Historiography on Venice	References	Deep learning, Word embeddings
He and Chen (2018)				
Modeling Citation Contexts	Life Sciences and Biomedical	PubMed Central Open Access Subset	Fulltext, metadata	Temporal citation embedding models
Nomoto (2018)				
Citation linking	Computational Linguistics	ACL Antology, CL-SciSumm	Fulltext, metadata	Neural networks
Mariani et al. (2019a,b)				
Analysing a research field, co-authorship, innovation, text reuse and plagiarism	Speech & Language Processing	NLP4NLP	Fulltext, metadata	Network analysis, terms frequency, time series prediction, text similarity
Valdez and Goodson				
Predicting bias in research	Health	PubMed, EbscoHost, Web of Science	Abstracts, metadata	Topic modeling
Kandimalla et al.				
Classifying scholarly papers	Computer Science, Physics	Web of Science	Abstracts	Neural networks
van Haastrecht et al.				
Systematic reviewing	Cybersecurity	Scopus, Web of Science, PubMed	Metadata	Backward snowballing, Active learning
Turki et al.				
Knowledge Graph Extraction	Bibliographic metadata	–	Metadata	–
Yamamoto et al.				
Identifying visual summaries	Computer Science	Semantic Scholar	Fulltext	Self-supervised learning, Transformer
Mariani et al.				
Analysing the evolution of a research field	Speech & Language Processing	NLP4NLP+5	Fulltext, metadata	Network analysis, terms frequency, time series prediction, text similarity

## Author Contributions

All authors listed have made a substantial, direct, and intellectual contribution to the work and approved it for publication.

## Funding

The work by IA was partly funded by the French ANR project InSciM Modeling Uncertainty in Science ANR-21-CE38-0003-01. The work by MB was partly funded by the French ANR project TheoScit Study of citation contexts for a construction of semantic relational indicators ANR-20-CE38-0003-01.

## Conflict of Interest

The authors declare that the research was conducted in the absence of any commercial or financial relationships that could be construed as a potential conflict of interest.

## Publisher's Note

All claims expressed in this article are solely those of the authors and do not necessarily represent those of their affiliated organizations, or those of the publisher, the editors and the reviewers. Any product that may be evaluated in this article, or claim that may be made by its manufacturer, is not guaranteed or endorsed by the publisher.

## Footnotes

^1^ https://www.frontiersin.org/research-topics/7043/mining-scientific-papers-nlp-enhanced-bibliometrics

^2^ https://www.frontiersin.org/research-topics/13388/mining-scientific-papers-volume-ii-knowledge-discovery-and-data-exploitation
